# Estimating lifetime risk of diabetes in the Chinese population

**DOI:** 10.1371/journal.pmed.1004053

**Published:** 2022-07-21

**Authors:** Fiona Bragg, Zhengming Chen

**Affiliations:** 1 Clinical Trial Service Unit & Epidemiological Studies Unit (CTSU), Nuffield Department of Population Health, University of Oxford, Oxford, United Kingdom; 2 Medical Research Council Population Health Research Unit (MRC PHRU), Nuffield Department of Population Health, University of Oxford, Oxford, United Kingdom

## Abstract

In this Perspective, Fiona Bragg and Zhengming Chen discuss the burden of diabetes in the Chinese Population.

The worldwide epidemic of diabetes continues to grow [1]. In China, the rise in prevalence has been notably rapid; about 12% of the adult population has diabetes [2], accounting for almost one quarter of cases worldwide [1] and representing a 10-fold increase over the last 3 to 4 decades. It is appropriate, therefore, that diabetes—both prevention and management—is a major focus of current health policy initiatives in China [3,4], and their success depends on reliable quantification of the burden of diabetes. Commonly used measures such as prevalence and incidence fail to capture excess mortality risks or differences in life expectancy in diabetes [5]. Moreover, they may be less easily interpreted by policy makers and affected individuals. Estimates of lifetime risks and life years spent living with diabetes in an accompanying study by Luk and colleagues provide a valuable new perspective on the burden of diabetes in the Chinese population [6].

The study used Hong Kong territory-wide electronic health records data for 2.6 million adults. Using a Markov chain model and Monte-Carlo simulations, Luk and colleagues estimated age- and sex-specific lifetime risks of diabetes (incorporating both clinically diagnosed and undiagnosed diabetes) and remaining life years spent with diabetes. Their findings showed a lifetime risk of 65.9% and 12.7 years of life living with diabetes for an average 20-year old with normoglycaemia. For an average 20-year old with prediabetes the corresponding estimates were 88.0% and 32.5 years, respectively. In other words, 6 out of 10 20-year olds with normoglycaemia and 9 out of 10 with prediabetes would be expected to develop diabetes in their lifetime. The estimated lifetime risks declined with increasing age and were higher among women than men at all ages, likely reflecting women’s higher life expectancy.

These estimated lifetime risks are striking and concerning. Moreover, they are notably higher than western population estimates [7–10], including those considering both diagnosed and undiagnosed diabetes [9,10]. An Australian study estimated that 38% of 25-year olds would develop diabetes in their lifetime [10]. Another study in the Netherlands reported 31.3% and 74.0% probabilities of developing diabetes in the remaining lifetime for individuals aged 45 years without diabetes and with prediabetes, respectively [9]. Diabetes incidence and overall mortality influence population lifetime risks. Differences in the glycaemic indicators used to identify undiagnosed diabetes may have contributed to differences between studies in diabetes incidence. In the study by Luk and colleagues, a combination of fasting plasma glucose (FPG), HbA1c levels and oral glucose tolerance testing (OGTT) was used, while in the Australian [10] and the Netherlands [9] studies, they used FPG/OGTT and mainly FPG, respectively. However, it is unlikely these differences would fully account for the large disparities seen in lifetime risk. Similarly, differences between life expectancy in Hong Kong (84.8 years), Australia (83.4 years), and the Netherlands (82.2 years) are too small to explain the differences. Interestingly, the high lifetime risks observed in Hong Kong were more comparable to those in the Indian population, estimated at 55.5% and 64.6%, respectively, among 20-year-old men and women [11]. The typical type 2 diabetes (T2D) phenotype in these Asian populations may partly explain their higher estimated lifetime risks. More specifically, T2D in both Chinese and Indian populations is characterised by onset among younger and less adipose individuals than typically observed in western populations, exacerbated by rapid urbanisation and associated unhealthy lifestyles [12].

However, aspects of Luk and colleagues’ study design may have overestimated lifetime diabetes risks. Chief among these is the data source used and associated selection bias. The Hong Kong Diabetes Surveillance Database includes only individuals who have ever had a plasma glucose or HbA1c measurement undertaken in a local health authority facility. Since measurement of glycaemic indicators is more likely among individuals at greater current or future risk of dysglycaemic states, this will have inflated estimates of lifetime risk and life years spent with diabetes. Although replication was undertaken by the study authors to address this bias in the smaller China Health and Retirement Longitudinal Survey (CHARLS) cohort, it does not fully allay these concerns, with modestly lower estimated lifetime diabetes risks in the CHARLS cohort, even after accounting for its higher mortality. A further limitation is their consideration of transition to dysglycaemic states as irreversible. Although data on long-term transition between glycaemic states are lacking, reversion from prediabetes (and less commonly diabetes) to normoglycaemia is well recognised, e.g., through lifestyle interventions [13].

Large-scale population-based cohort studies could valuably address many of the limitations described [14]. Furthermore, lifetime risks are, by definition, population-based and represent the risk of an average person in the population, limiting their value for communicating long-term disease risks to specific individuals. However, the extensive phenotyping (e.g., adiposity) characteristic of many large contemporary cohorts [14] would facilitate incorporation of risk factors into lifetime risk estimates, enhancing their relevance to individuals. Previous studies have found greater lifetime risks of diabetes associated with adiposity [9,11], and this approach could be extended to incorporate other established, as well as more novel (e.g., genetic), risk factors. This is arguably of particular relevance to later-onset chronic conditions, such as T2D, in which changes in risk factors during middle age can influence lifetime risks. A valuable extension of Luk and colleagues’ study will be estimation of risk factor specific lifetime diabetes risks for the Chinese population.

Importantly, the limitations described do not detract from the enormity and importance of the challenge diabetes poses for China, including Hong Kong, and the estimates presented by Luk and colleagues provide valuable impetus for action. The disease burden insights can inform treatment programmes and enhance understanding of current and future impacts of diabetes and associated complications on the healthcare system. Moreover, T2D is preventable, and arguably, the greatest value of these estimated lifetime risks is in highlighting the need for, and informing the planning and provision of, diabetes primary prevention programmes. This includes identification of high-risk individuals, such as those with prediabetes, who are most likely to benefit from prevention interventions. However, the magnitude of the estimated lifetime diabetes risks, including among the large proportion of the population in a normoglycaemic state, additionally demonstrates the need for population-level prevention approaches, including environmental, structural, and fiscal strategies. Without such actions, the individual and societal consequences of diabetes for present and future generations in Hong Kong, as well as mainland China, will be immense.

## Abbreviations

CHARLS: China Health and Retirement Longitudinal Survey
FPG: fasting plasma glucose
OGTT: oral glucose tolerance testing
T2D: type 2 diabetes
